# The East Africa Consortium for human papillomavirus and cervical cancer in women living with HIV/AIDS

**DOI:** 10.1080/07853890.2022.2067897

**Published:** 2022-05-06

**Authors:** Y. Tong, E. Orang’o, M. Nakalembe, P. Tonui, P. Itsura, K. Muthoka, M. Titus, S. Kiptoo, A. Mwangi, J. Ong’echa, R. Tonui, B. Odongo, C. Mpamani, B. Rosen, A. Moormann, S. Cu-Uvin, J. A. Bailey, C. I. Oduor, A. Ermel, C. Yiannoutsos, B. Musick, E. Sang, A. Ngeresa, G. Banturaki, A. Kiragga, J. Zhang, Y. Song, S. Chintala, R. Katzenellenbogen, P. Loehrer, D. R. Brown

**Affiliations:** a Indiana University School of Medicine, Indianapolis, IN, USA; b Moi University, Eldoret, Kenya; c Infectious Diseases Institute, Makerere University, Kampala, Uganda; d Maseno University, Kisumu, Kenya; e Kenya Medical Research Institute, Eldoret, Kenya; f Beaumont Gynecology Oncology, Royal Oak, MI, USA; g University of Massachusetts Chan Medical School, Worcester, MA, USA; h Brown University, Providence, RI, USA; i AMPATH, Eldoret, Kenya; j Indiana University Fairbanks School of Public Health, Indianapolis, IN, USA

**Keywords:** HPV, HIV, Kenya, Uganda, cervical cancer

## Abstract

The East Africa Consortium was formed to study the epidemiology of human papillomavirus (HPV) infections and cervical cancer and the influence of human immunodeficiency virus (HIV) infection on HPV and cervical cancer, and to encourage collaborations between researchers in North America and East African countries. To date, studies have led to a better understanding of the influence of HIV infection on the detection and persistence of oncogenic HPV, the effects of dietary aflatoxin on the persistence of HPV, the benefits of antiretroviral therapy on HPV persistence, and the differences in HPV detections among HIV-infected and HIV-uninfected women undergoing treatment for cervical dysplasia by either cryotherapy or LEEP. It will now be determined how HPV testing fits into cervical cancer screening programs in Kenya and Uganda, how aflatoxin influences immunological control of HIV, how HPV alters certain genes involved in the growth of tumours in HIV-infected women. Although there have been challenges in performing this research, with time, this work should help to reduce the burden of cervical cancer and other cancers related to HIV infection in people living in sub-Saharan Africa, as well as optimized processes to better facilitate research as well as patient autonomy and safety.

**KEY MESSAGES**
- The East Africa Consortium was formed to study the epidemiology of human papillomavirus (HPV) infections and cervical cancer and the influence of human immunodeficiency virus (HIV) infection on HPV and cervical cancer.
- Collaborations have been established between researchers in North America and East African countries for these studies.
- Studies have led to a better understanding of the influence of HIV infection on the detection and persistence of oncogenic HPV, the effects of dietary aflatoxin on HPV detection, the benefits of antiretroviral therapy on HPV persistence, and the differences in HPV detections among HIV-infected and HIV-uninfected women undergoing treatment for cervical dysplasia by either cryotherapy or LEEP.

## Introduction

The East Africa Consortium (the Consortium) was formed in 2014 for studies of the epidemiology of human papillomavirus (HPV) infections and cervical cancer in Kenyan women infected with the human immunodeficiency virus (HIV). The Consortium was established in within the Academic Model Providing Access to Healthcare (AMPATH), a partnership between Moi University School of Medicine (MUSOM), Moi Teaching and Referral Hospital (MTRH), and a group of North American medical schools led by Indiana University [1]. The purpose of this manuscript is to describe the formation of the Consortium, research projects, capacity building, and results to date. The initial version (Version 1) was formed in 2014 and included researchers from the USA and MUSOM (Kenya). The second version (Version 2) was formed in 2021 and expanded to include the Uganda Cancer Institute (UCI) and Makerere University in Uganda. A brief overview of the epidemiology of HPV infection, cervical cancer, the role of HIV infection will be presented followed by descriptions of Versions 1 and 2 of the Consortium.

## The epidemiology of HPV and cervical cancer among East African women

Each year, cervical cancer is responsible for nearly 300,000 deaths worldwide; 90% of these deaths occur among women living in low- and middle-income countries [2]. Cervical cancer is the most common malignancy among women living in sub-Saharan Africa (SSA) [3,4]. In 2018, the age-standardized incidence and mortality rates for cervical cancer were 40 and 30 per 100,000 per year for women living in East Africa compared with 6.4 and 1.9 per 100,000 per year for women living in North America [5,6]. Also, the incidence of cervical cancer has been increasing in SSA; in western Kenya for example, there has been a 9.5% increase from 2002 to 2016 [7].

Oncogenic types of human papillomaviruses (“high-risk” HPV or HR-HPV) are the causative agents of cervical cancer [8]. Of the 13 HR-HPV types classified by International Agency for Research on Cancer (IARC) as being carcinogenic, HPV types 16 and 18 cause approximately 70% of all cervical cancer worldwide [9,10]; other HR-HPV types cause the remainder of cervical cancers. Detection of HR-HPV over a one or two-year period (persistence) is associated with a markedly increased risk of cervical cancer [11–14].

Although many wealthy countries have controlled the spread of HIV infections to a large part, the HIV epidemic continues in sub-Saharan Africa. Although there have been gains in Africa, the epidemic continues. SSA contains 70% of the world’s diagnosed cases of HIV/AIDS, with countries in East Africa such as Kenya at its epicentre (7% prevalence) [15]. Infection with HIV greatly accelerates the natural history of HPV infection [16–19]. Women who are HIV-infected have a higher relative risk of developing cervical, anal, and other cancers caused by HPV infection compared with HIV-uninfected women [20–22]. The incidence of cervical cancer has not changed significantly since antiretroviral therapy (ART) was introduced for the treatment of HIV infection, possibly because of pre-existing HPV infections that occurred (and progressed) before ART was administered [23,24]. The burden of HPV-related cancers can be expected to increase in HIV-infected patients, given the prolongation of life with the use of ART, leading to longer duration of HPV persistence and accumulation of mutations that contribute to carcinogenesis. Although HIV infection accounts for much of the high incidence and mortality of cervical cancer in Kenyan women, other cofactors are important, as HIV-uninfected Kenyan women also suffer from a high burden of cervical cancer. These factors are yet to be identified but may include environmental factors such as indoor smoke exposure, dietary toxins such as aflatoxin, micronutrient deficiency, and others. It is biologically plausible that these environmental factors interact with HPV to increase the risk of cervical cancer.

Cervical cancer develops slowly, and although the natural history is accelerated in women living with HIV/AIDS, it is possible to detect precancerous lesions by screening. In addition, effective vaccines have been available since 2006 that prevent infection with HR-HPV [25]. Thus, cervical cancer should be preventable with a combination of effective screening of all adult women and vaccination of all children and adolescents against HPV infection. Unfortunately, screening programs for cervical cancer reach a limited number of women in poor countries, where a vast majority of cases of this malignancy occur. Only 5% of Kenyan women are regularly screened for cervical cancer and only 14% have *ever* been screened due to obstacles that include lack of clinics that provide the service, travel to clinics, and other factors [26].

The need for cost-effective screening approaches for cervical cancer prevention in low-income countries has led to widespread use of visual assessment of the cervix following the application of 5% acetic acid, a test known as visual inspection with acetic acid, or VIA [27–32]. Although relatively inexpensive, VIA has only modest sensitivity (∼60 to 70%) and specificity (∼50%) for detection of cervical intraepithelial neoplasia (CIN), which may be pre-invasive lesions [29]. Despite these limitations, VIA is likely to be better than no screening of any kind. Cervical cancer screening is done in wealthy countries in clinics using clinician-obtained samples with cytological testing (Pap smear) or/and molecular methods (testing for HR-HPV). Development of advanced methods for cervical cancer screening has been slow in low-income countries due to the need for infrastructure, training, and high costs. Thus, it is critical we move beyond VIA and that more sensitive and specific biomarkers of early disease and progression are identified, particularly those that may be specific including markers in women infected with HIV.

## The Consortium, version 1: “HPV and cervical cancer in Kenyan women with HIV/AIDS”

Version 1 of the Consortium was entitled “HPV and Cervical Cancer in Kenyan Women with HIV/AIDS” and funded by the NCI from 2014 to 2019. The core objectives of Version 1 were to better understand the natural history of oncogenic HPV infections in HIV-infected Kenyan women, and to identify potentially modifiable factors associated with progression of oncogenic HPV infection to cervical cancer. A major objective has been capacity building. Version 1 was approved by the ethics review boards of Moi Teaching Referral Hospital (MTRH) and Moi University, Eldoret, Kenya, the Kenya Medical Research Institute’s Scientific and Ethics Review Unit (KEMRI-SERU) and by the Institutional Review Boards of Indiana University School of Medicine and Brown University.

### Projects in version 1

Two research projects comparing HIV-infected and HIV-uninfected women were included in Version 1. Project 1 was entitled “Modifiable factors for HPV persistence in Kenyan women”. Project 2 was entitled “Results of cryotherapy or loop electrosurgical excision procedure (LEEP) among HIV-infected and HIV-uninfected women in western Kenya”.

#### Version 1, project 1 “modifiable factors for HPV persistence in Kenyan women”

The first aim of Project 1 was to recruit a balanced (HIV-infected and HIV-uninfected) cohort of women without evidence of cervical disease (based on VIA), and to describe the frequency and distribution of oncogenic HPV in cervical samples. The second aim was to identify potentially modifiable behavioural and biological factors associated with two outcome variables [5]: persistence of oncogenic HPV, and [33] cervical dysplasia.

#### Brief description of methods and results for project 1

A cohort was enrolled in 2015 to 2016 consisting of 116 HIV-infected and 106 HIV-uninfected women aged 18 to 45 years, with normal VIA that day at the AMPATH Cervical Cancer Screening Program (CCSP), located at MTRH, in Eldoret, Kenya [34]. Studies conducted as part of in Project 1 indicated that oncogenic and non-oncogenic HPV types were detected more often among HIV-infected women compared with HIV-uninfected women, and that persistent HPV (one-year persistence or two-year persistence) was more often detected among HIV-infected women than HIV-uninfected women [35]. In addition, a longer duration of ART use was associated with significantly reduced risk of HR-HPV detection and persistence [36]. Lastly, exposure to dietary aflatoxin was significantly associated with detection of oncogenic HPV types [37]**.**

#### Version 1, project 2 “results of cryotherapy or loop electrosurgical excision procedure (LEEP) among HIV-infected and HIV-uninfected women in Western Kenya”

Three aims were included in Project 2. The first aim was to assess the results of cryotherapy or LEEP among HIV-infected and HIV-uninfected women in Western Kenya over 36 months of follow-up. The second aim was to assess the risk factors associated with treatment failures among HIV-infected and HIV-uninfected women undergoing cryotherapy or LEEP. The third aim was to describe the frequency and distribution of HR-HPV types among HIV-infected and HIV-uninfected women undergoing cryotherapy or LEEP over 36 months of follow-up.

#### Brief description of methods and results for project 2

Women between the ages of 18 and 45 years with an abnormal VIA examination were tested for HPV and treated with cryotherapy for lesions not suspicious of cancer [38]. Colposcopy and biopsy were performed in women with suspicious lesions, and LEEP was performed if biopsy showed CIN2/3. HIV-infected women requiring LEEP were more likely to have HR-HPV types and to be infected with multiple HR-HPV types compared with those undergoing cryotherapy; there were no such differences in HPV types identified among HIV-uninfected women. Additional studies will assess the efficacy of cryotherapy and LEEP as treatment for CIN lesions, and determine the risk factors associated with treatment failures among HIV-infected and HIV-uninfected women undergoing these treatment modalities.

## The consortium, version 2 (“the East Africa Consortium for HPV and cervical cancer in women living with HIV/AIDS”)

The main objectives of Version 2 of the Consortium are 1) to better elucidate the natural history of HPV infection and cervical cancer in HIV-infected women living in East Africa, 2) to study the persistence and progression of CIN after LEEP, and 3) to identify new viral and cellular biomarkers that will assist in screening, triage, and treatment of precancerous cervical lesions (CIN2/3) and cervical cancer. The central hypothesis of Version 2 is that the incidence, persistence, and spectrum of HR-HPV are substantially greater in HIV-infected East African women, and that this explains the higher incidence of cervical neoplasia. We further hypothesize that these and other modifiable factors, such as aflatoxin ingestion, disproportionately and adversely influence outcomes of therapies in HIV-infected women.

The overall specific aims for Version 2 are 1) to establish a sustainable research infrastructure for an international partnership to conduct impactful research in HPV and cervical cancer in women living with HIV/AIDS, 2) to design and execute three integrated projects that advance the knowledge of the environmental and biologic factors leading to cervical cancer in East Africa, and 3) to increase the research workforce capacity in East Africa through mentoring, training programs and targeted pilot projects. The goal is to establish a dedicated team of cancer-focussed East African investigators who embody a culture of translational and collaborative research. To accomplish this, we bring together two respected research programs: AMPATH in Kenya and the Infectious Disease Institute (IDI) in Uganda.

Version 2 was approved by the ethics the review boards at Moi Teaching Referral Hospital (MTRH) and Moi University, the Kenya Medical Research Institute’s Scientific and Ethics Review Unit (KEMRI-SERU), the Uganda Cancer Institute (UCI), Makerere College of Health Sciences, and by the Institutional Review Boards of Indiana University School of Medicine and Brown University.

### Addition of Uganda to the consortium

The NCI supported the natural expansion of the consortium to include a second country to be added to Version 2. Uganda was added because of its proximity to Kenya, previous partnerships in research and training, and complementary research capacity already existing in Uganda. Facilities, equipment, and support required for cervical cancer screening, testing, biopsy, and treatment were available, as well as core facilities providing HIV testing, CD4-cell counts, HIV viral load, and pathology services. The UCI is a public facility that serves as the national referral centre for all cancer cases in Uganda and other neighbouring countries. UCI is one of the Uganda Ministry of Health facilities and is also affiliated to Makerere University College of Health Sciences and the Mulago National Referral Hospital, the teaching hospital for the medical school. In addition, the Infectious Diseases Institute (IDI), affiliated with Makerere College of Health Sciences, was deemed a good fit for Version 2 of the U54 project. The mission of the IDI is to build African health systems capacity for the delivery of sustainable, high quality care as well as prevention of HIV/AIDS and related conditions through training, research, and advanced clinical services. The IDI currently offers training in HIV/AIDS, malaria, pharmacy, and laboratory services.

#### Specific projects in version 2

Three projects are included in Version 2; all three projects are focussed on the study of cervical cancer in HIV-infected women living in Eastern Africa.

**Version 2, Project 1** is entitled “Preventing cervical cancer in HIV-infected Kenyan and Ugandan women”. The long-term objective of Project 1 is to help eradicate cervical cancer in HIV-infected Kenyan and Ugandan women, and contains two Specific Aims. Aim 1 is to evaluate HR-HPV DNA testing of self-collected vaginal swabs combined with VIA in screening for cervical cancer in HIV-infected women living in Kenya or Uganda. This aim was designed to address multiple questions. First, what are differences in detection of biopsy-proven CIN2/3 between all HIV-infected women living in Kenya or Uganda with both HR-HPV DNA positivity and VIA abnormality compared with HIV-infected women with only one or neither of these tests positive? Second, is there consistency in detection of biopsy-proven CIN2/3 among women living in either Kenya or Uganda who have both HR-HPV DNA positivity and VIA abnormality compared to those with only one or neither of these tests positive?

The second aim 2 of Project 1 is to determine if aflatoxin is a risk factor for cervical cancer among HIV-infected Kenyan and Ugandan women. This aim is designed to address the following questions. First, does exposure to aflatoxin increase the risk of HR-HPV detection, HR-HPV persistence, and cervical dysplasia in Kenyan/Ugandan women? Second, is aflatoxin detection/concentration in plasma associated with higher HIV viral load measurements and lower CD4-cell counts in HIV-infected women?

#### Description of methods for project 1

Kenyan and Ugandan women 21 to 60 years of age will be invited to enroll in a three-year longitudinal study at two sites: the CCSP at MTRH in Kenya, and the UCI and IDI Clinics in Uganda. Kenyan women living in or within 30 km of Eldoret who present for cervical cancer screening at the CCRP, and Ugandan women who live within 30 km of Mulago who present for cervical cancer screening at the UCI/IDI clinic will be asked to participate in the study are between the ages of 21 and 60 years, and are willing to return for four total visits over a three-year total period. Exclusion criteria will include current pregnancy, inability to consent due to mental or physical disability, or a medical illness that has rendered the patient unable to attend annual visits. Details of sexual history are captured in the questionnaires used in the study; no women are excluded based on sexual history alone.

A total of 240 women will be enrolled: 60 HIV-infected and 60 HIV-uninfected women in Kenya (*n* = 120) and 60 HIV-infected and 60 HIV-uninfected women in Uganda (*n* = 120).

Women will be instructed on the purpose and technique of self-collection of vaginal swabs. Swabs will be collected and delivered to the laboratory and tested for HR-HPV DNA using the Roche Cobas assay, which provides a yes/no answer for any of 14 high-risk HPV types, and also provides information on specific detection of HPV types 16 and 18. All women will also undergo VIA, the standard screening method utilized in Kenya. A blood sample will be taken for measurement of aflatoxin, a carcinogenic and immunosuppressive mycotoxin found in corn, the main source of calories for women in Kenya and Uganda. Exclusion criteria will include current pregnancy, inability to consent due to mental or physical disability, or a medical illness that has rendered the patient unable to attend annual visits and inability to fully visualize the squamo-columnar junction of the cervix during the VIA examination.

HPV DNA test results will not be used to triage women for VIA, and all women will undergo VIA in this study, since this is the current standard of care in sub-Saharan Africa; treatment is based on VIA results. At enrolment, all women with abnormal VIA examinations will undergo cervical biopsy of the lesion. In addition, at enrolment, all HIV-infected women with normal VIA will undergo cervical biopsy at two random sites, because a goal of the project is to determine detection of biopsy-proven CIN2/3 in women with positive HPV DNA combined with abnormal VIA compared with those either test individually positive/abnormal. For HIV-uninfected women, only those with abnormal VIA examinations will undergo cervical biopsy of the lesion; HIV-uninfected women with normal VIA will not undergo biopsy, because it has already been established in studies that HPV DNA is superior in sensitivity and other parameters compared with VIA in these women. Women with abnormal VIA examinations will be treated according to established local algorithms, regardless of HIV status.

**Version 2, Project 2** is entitled “Understanding CIN2+ among HIV-infected women after LEEP: An epidemiological and immunohistochemical study”. The objective of Project 2 is to better understand CIN2/3 lesions after LEEP in HIV-infected women, and will examine the concept that if cancer-susceptible junctional cells at the squamo-columnar junction are destroyed, they do not regenerate regardless of the gross visual appearance of the healed tissue at the cervix after excision. There are three Specific Aims in Project 2. The first is to assess the incidence and risk factors for “recurrent” CIN2/3 after LEEP among HIV-infected women. Specific Aim 2 is to determine baseline HR-HPV types (in tissue) among HIV-infected women with CIN2/3 undergoing LEEP compared with the HR-HPV types with “recurrent” cervical lesions after a second LEEP. Specific Aim 3 is to determine the immunohistochemical differences between the initial and repeat LEEP specimens among HIV-infected women with “recurrent” CIN2/3.

#### Description of methods for project 2

This observational cohort study will involve 300 HIV-infected, Kenyan and Ugandan women diagnosed with CIN2/3 who are undergoing LEEP, who are 18 to 60 years of age, and who express a willingness to return for long-term follow-up. Exclusion criteria will include prior history of CIN2/3, cervical cancer, signs or symptoms of a current STI, currently pregnant, inability to consent due to mental or physical disability, or an intervening medical illness that has rendered the patient unable to understand consent or to attend follow-up visits. Details of sexual history are captured in the study questionnaires, and no woman will be excluded based on sexual history alone. Women will be eligible for LEEP if an acetowhite lesion of the cervix is observed that is associated with the transformation zone and has indefinite margins, if the lesion covers ≥75% of the transformation zone, if the lesion is not seen in its entirety and/or disappears into the endocervical canal, and if there is no clinical evidence of cervical cancer.

At each visit, participants will complete a face-to-face interview by a research associate and fill out a brief questionnaire in either English or Swahili. Demographic information as well as a full medical history (CD4 cell counts, plasma viral load if available, WHO disease stage, ART, sexual history, STI history, and other medical co-morbidities) will also be obtained. Follow-up visits will be based on the current standard of care (six months after LEEP). All patients will undergo colposcopy of the cervix and vagina with biopsy if appropriate. Those with recurrent CIN2/3 will undergo LEEP. Those with cervical cancer or other identified cancers will be referred for treatment. For patients with no lesions on colposcopy, follow-up will continue every six months.

HPV testing of cervical tissue will be done by Type Seq Linear Array at the National Cancer Institute (USA). Immunohistochemistry will be performed on cervical biopsy samples (the original LEEP specimen and the repeat LEEP specimen) to detect p16ink4, ki67, and five embryonic squamo-columnar junction specific antibodies (Keratin 7, ARG2, CD63, MMP7, and GDA). These markers will help demonstrate the presence of unique cuboidal cells that might be responsible for HPV-related cancer.

**Version 2, Project 3** is entitled “Determining biological and viral factors associated with clinical progression of cervical dysplasia in HIV-infected women”, and has two aims. The first is to identify uniquely expressed functional host cell markers that are fundamental to HR-HPV infection and cervical dysplasia, and to stratify risk in women who are HIV-infected or HIV-uninfected based on these biomarkers. This aim is designed to determine if HR-HPV co-opts and dysregulates host cell pathways that cannot be resolved and normalized in women who are HIV-infected, and if the synergy of HPV and HIV is founded in HR-HPV downregulating innate immunity and HIV downregulating cell-mediated immune-surveillance. Aim 2 is to create a baseline map of HPV 16 variants found in Kenyan and Ugandan women. This aim is designed to identify if any specific HPV 16 variants in Eastern Africa are associated with cervical cancer progression.

#### Description of methods for project 3

For Aim 1, blood samples from Kenyan and Ugandan women will be examined for proteases that are known to be altered by HIV and in cervical cancer patients. Serum/plasma from HIV-infected and HIV-uninfected women will be mixed with biotinylated detection antibodies. The sample/antibody mixture will then be incubated with a capture array. Any protein/detection antibody complexes that are present will be bound by a cognate immobilized capture antibody on a membrane, then detected and quantified using chemiluminescent detection reagents. Comparisons will then be made between samples from HIV-infected and HIV-uninfected women. In addition, quantification of proteases (Cathepsin S, L, B, D and E) and protease inhibitors (Cystatins, Serpins and TIMPs) in blood samples will be performed by ELISA to look for differences between HIV-infected and HIV-uninfected women.

Cervical biopsies collected from women with abnormal VIA examinations will be processed for immunohistochemical staining (NFX1-123, Ki67, Notch1, Keratin 1, and Involucrin). The staining intensity for each protein of interest will be quantified. In addition, RNA sequencing will be used to quantify gene expression differences in cervical biopsies including hTERT, NFX1-123, Notch1, SLPI, innate immune pathway and target genes, and cathepsins. Analysis for differences in gene expression between samples from HIV-infected and HIV-uninfected women will be determined by standard curve analyses.

Cervical swabs will also be collected for gene expression profiling of host biomarkers that may differ between HIV-infected and HIV-uninfected women. We will follow a specific process to collect these endocervical swabs for two functions: total RNA will be extracted from cervical cells for quantitative real-time PCR (qPCR) studies and cells will be used for single cell sequencing. For qPCR studies, total RNA will be purified from swab samples, and cDNA will be generated using random hexamer primers and reverse transcriptase. qPCR will be performed, and analyses for differences in gene expression (hTERT, NFX1-123, Notch1, SLPI, innate immune pathway and target genes, and cathepsins) will be executed by standard curve analyses. Single cell RNA sequencing will also be explored using approximately 1,000 to 10,000 targeted cells per endocervical swab sample. Methods will include SeqWell which allow for in-country capture from fresh swabs. We will examine for the single cells for differences in cellular populations and cell state between HIV-infected and HIV-uninfected woman.

For Aim 2, we will determine if there are specific HPV 16 variants in women who are HIV-infected compared with HIV-uninfected women. Women with HPV 16 infections will be identified, and whole genome sequencing of HPV 16 isolates will be performed using two complementary methods: molecular inversion probes (MIPs) and long-range PCR. A set of molecular inversion probes and long-range PCR primers tiling across the entire HPV genome be developed to amplify and capture the HPV 16 genome. At the end of this process, the HPV 16 genome in its entirety will be assembled. Specific variations of the HPV 16 genome will be assessed for association with HIV-infected or HIV-uninfected, and correlated to specific mRNA and protein expression differences.

## Description of cores

Four cores developed for Version 1 were continued in Version 2. Responsibilities for each of these cores were shared between North American, Kenyan, and Ugandan investigators to foster interactions and to support further leadership development of our Kenyan and Ugandan colleagues. These cores are described below.

### The administration and coordination core (ACC)

The ACC was organized to provide administrative and leadership support for all projects and for the other cores. This core was assisted by an External Advisory Group to provide input regarding the dissemination and integration of findings of the consortium.

### The biostatistics and data management core (BDMC)

The BDMC has provided research collaboration for projects, management of data and oversight of informatics needed for each project, and training in the form of both modules and on-the-job mentoring. Specifically, the BDMC individual goals were to 1) Provide faculty level collaboration on each project for informatics, data management, study design, data analysis, interpretation of results, and preparation of manuscripts and abstracts, 2) Provide support for creation and maintenance of project-specific databases, to provide support for converting databases between platforms (e.g. from ACCESS to SAS); to facilitate access to the AMPATH Medical Record System (AMRS) as needed for each project, 3) To integrate data from various sources, and to create a centralized database for the U54 group, 4) Provide programming support for basic and advanced data analysis, including generation of preliminary reports, model fitting for investigator-led studies, and development of software code for specific analyses, 4) Ensure quality control and reproducibility of all data analyses by creating project-specific archives that contain: a final analysis database for each specific project; the programs used for creation of variables, including combinations of items into scales; data analysis (written as scripts for the appropriate software package), and subsequent versions of abstracts and manuscripts, and 5) Serve as a locus for didactic and on-the-job training for core members in Kenya on biostatistics, study design, and reproducible research in the form of modules and seminars, for the purpose of building intellectual capacity in biostatistics and informatics. For Version 2, a coordination was developed between the International Epidemiology Databases to Evaluate AIDS (IeDEA) Collaboration and the Consortium [33]. The East Africa IeDEA Regional Consortium (EA-IeDEA) has established a vast data infrastructure in East Africa over the past 15 years. The BDMC is embedded within the IeDEA-EA Regional Data Centre to address the research aims of all three projects in the Consortium. IeDEA-EA has access to all clinical data collected as part of routine HIV care and treatment of the women who will be involved in our study. These data will be merged with data from the REDCap databases created for the prospective studies undertaken in this proposal, thus leveraging a large amount of resources and data infrastructure developed at AMPATH and the IDI, as well as experience with data collected as part of routine HIV care and treatment.

### The mentoring and career development core (MCDC)

The MCDC serves to enhance the research training capacity in HIV-related malignancies. Trainees will be chosen based on submitted proposals and educated through short courses on oncology research, and leadership seminars were provided focussing on epidemiology and data management. Trainees will design and carry out pilot projects related to the main projects described above, and will be encouraged to write and submit publications and submit projects for extramural funding based on preliminary findings. An important component includes a focus on career development and advancement at the respective institution of the trainee. This includes online modules and group discussions based on the Afya Bora Fellowship in Global Health Leadership program http://www.afyaboraconsortium.org/index.html. To date, five manuscripts have been published in peer-reviewed journals by trainees working within the MCDC [39–43].

### The translational biology core (TBC)

The TBC provides support for all projects by providing labelling and storage of samples collected from women in the study, transportation to appropriate laboratories, STI testing, HPV analysis, and providing space for young physician scientists, graduate students and technicians to enhance their laboratory expertise. During Version one of the U54 program, a Biobank was developed at the Chandaria Oncology and Chronic Disease Centre in Eldoret, Kenya. The Biobank is now housed in secure space and is equipped to process and store specimens of varying types. The Biobank also employs a laboratory information system to catalog samples and to track shipments of specimens to other investigators. As part of Version 2, the Biobanks at AMPATH in Eldoret, Kenya and at the IDI in Kampala, Uganda will collaborate to facilitate the transfer of technologies between sites, and to provide a more robust training environment for cancer research in East Africa.

## Discussion: challenges and conclusions

The Consortium was designed to allow a joint effort between research centres in North America and sub-Saharan Africa to study the epidemiology of HPV infections and cervical cancer in HIV-infected and HIV-uninfected women living in Kenya or Uganda. Version 1 of the overall program entitled “AMPATH Oncology Institute: HPV and Cervical Cancer in Kenyan Women with HIV/AIDS” was funded by the NCI from 2014 to 2019. Considerable progress was made in Version 1 in building collaborations between the participating Universities. The research studies in Version 1 led to a better understanding of the influence of HIV infection on detection and persistence of oncogenic HPV, the effects of dietary aflatoxin on persistence of oncogenic HPV, the benefits of ART on detection and persistence of oncogenic HPV, and the differences in HPV detections among HIV-infected and HIV-uninfected women undergoing treatment for cervical dysplasia by either cryotherapy or LEEP. The studies outlined in Version 2 will build on these accomplishments. We hope to better determine how HR-HPV testing fits into a cervical cancer screening program in Kenya and Uganda, how aflatoxin influences immunological control of HIV, how HPV alters certain genes involved in growth of tumours in HIV-infected women, and biomarkers involved in tumour progression can be identified.

There have been significant challenges in performing this research that will now involve three countries. Obtaining approvals from Ethics Committees was a challenge due to nuances between North American and African countries, and country-specific policies between Kenya and Uganda. This obstacle has led to discussions on how submissions and approvals can be respectfully harmonized between North America and African institutions. With time, this should lead to more optimized processes better facilitating research as well as patient autonomy and safety.

In conclusion, the Consortium, formed in 2014 for studies of the epidemiology of HPV infections and cervical cancer in HIV-infected and HIV-uninfected Kenyan women has facilitated the training of scientists and productive collaborative research between our countries. The second version of the Consortium will continue this work. We believe that such collaborations are critically important in reducing the burden of cervical cancer and other cancers related to HIV infection in people living in sub-Saharan Africa.

## Data Availability

The data that support the findings of this study are available from the corresponding author, [DB], upon reasonable request.
